# Endoscopic Ultrasound-Guided Pancreatic Interstitial Laser Ablation Using a Cylindrical Laser Diffuser: A Long-Term Follow-Up Study

**DOI:** 10.3390/biomedicines10112895

**Published:** 2022-11-11

**Authors:** Jungnam Lee, Youjeong Seo, Van Gia Truong, Hye Jung Jeong, Jung-Hyun Lim, Seonghee Lim, Hyun Wook Kang, Jin-Seok Park

**Affiliations:** 1 Digestive Disease Center, Department of Internal Medicine, Inha University College of Medicine, Inha University Hospital, Incheon 22332, Korea; 2 Department of Pathology, Inha University College of Medicine, Inha University Hospital, Incheon 22332, Korea; 3 Tecure, Inc., 45, Yongso-ro, Nam-gu, Busan 48513, Korea; 4 Industry 4.0 Convergence Bionics Engineering, Pug National University, Busan 48513, Korea; 5 Department of Biomedical Engineering and Marine, Integrated Biomedical Technology Center, Pukyong National University, Busan 48513, Korea

**Keywords:** cylindrical interstitial laser ablation, EUS, locally advanced pancreatic cancer, swine model

## Abstract

Background and Aims: Local ablative treatment is another option for improving outcomes and has been evaluated for locally advanced pancreatic cancer. We previously suggested endoscopic ultrasound (EUS)-guided interstitial laser ablation using a cylindrical laser diffuser (CILA) might be a feasible therapeutic option based on experiments performed on pancreatic cancer cell lines and porcine model with a short follow-up (3 days). The aim of this study was to investigate the safety of EUS-CILA performed using optimal settings in porcine pancreas with a long-term follow-up (2 weeks). Methods: EUS-CILA (laser energy of 450 J; 5 W for 90 s) was applied to normal pancreatic tissue in porcine (*n* = 5) under EUS guidance. Animals were observed clinically for 2 weeks after EUS-CILA to evaluate complications. Computed tomography and laboratory tests were carried out to evaluate safety. Two weeks after EUS-CILA, all pigs were sacrificed, and histopathological safety and efficacy evaluations were conducted. Results: EUS-CILA was technically successful in all five cases. No major complications occurred during the follow-up period. Body weight of porcine did not change during the study period without any significant change in feed intake. Animals remained in excellent condition throughout the experimental period, and laboratory tests and computed tomography (CT) scans provided no evidence of a major complication. Histopathological evaluation showed complete ablation in the ablated area with clear delineation of surrounding normal pancreatic tissue. Mean ablated volume was 55.5 mm^2^ × 29.0 mm and mean ablated areas in the pancreatic sections of the five pigs were not significantly different (*p* = 0.368). Conclusions: In conclusion, our experimental study suggests that EUS-CILA is safe and has the potential to be an effective local treatment modality. No major morbidity or mortality occurred during the study period. Further evaluations are warranted before clinical application.

## 1. Introduction

Pancreatic cancer has a dismal prognosis and, in 2020, it remained one of the deadliest cancers with approximately 496,000 new cases and 466,000 deaths worldwide [1]. Due to its increasing incidence and limited treatment options, pancreatic cancer was estimated to become the second leading cause of cancer-related death in 2030 [2]. Locally advanced pancreatic cancer (LAPC) is a difficult disease to treat. Improved preoperative therapy has been attempted in candidates previously considered unresectable for resection. However, the majority of patients with LAPC do not undergo surgical resection even after months of systemic chemotherapy [3,4]. Given this situation, effective new treatment strategies would have considerable impact, and thus another option, such as local treatment, is needed for advanced pancreatic cancer, especially LAPC.

Local treatments such as radiotherapy might provide readily available options that help improve local control and provide patients with a break from systemic chemotherapy. However, severe toxicity during and/or after concurrent chemoradiotherapy is a major limitation [5]. Thus, non-radiotherapy-based methods of local ablation have been developed and attempted [6,7]. Despite the lack of randomized controlled trials, these local treatments are increasingly being applied in combination with systemic chemotherapy in patients with LAPC [8].

Laser ablation (LA) is a type of local ablation and has the advantage of killing cancer cells while preserving normal tissue. LA has been evaluated for the minimally invasive treatments of thyroid, liver, and prostate cancer and, when minimally invasive, reduces pain and recovery times [9]. In addition, LA involves the use of flexible, small fibers, which enable the treatment of tumors in deep organs and high-risk, difficult-to-reach areas. However, conventional laser fibers have several shortcomings. In one study, it was concluded that a conventional LA diffuser can cause excessive thermal injury around target lesions, carbonized deposits to form around the diffuser tip, and result in non-uniform ablation [10]. In another study, peripancreatic fluid collection was found after LA was performed using a conventional unidirectional laser fiber [11].

The cylindrical laser diffuser was developed to address the limitations of unidirectional lasers and has been reported to be effective and safe in a xenograft mouse model of pancreatic cancer [12]. The developed cylindrical diffuser also produced uniform tissue necrosis and exhibited a positive relationship between delivered energy and necrotic area. In addition, we previously reported successful results for pancreatic interstitial laser ablation using a cylindrical laser diffuser (CILA) in a porcine model and that no definite post-procedural complications were encountered. However, previous studies have reported the efficacies and safety of CILA in the short-term (3 days). Thus, we considered a study was required to address long-term safety considerations because the pancreas is required for glucose, protein, and fat metabolism and for controlling endocrine and exocrine functions. Furthermore, ablative treatment could result in damage to surrounding normal tissues and adjacent organs such as duodenum and major vessels. Thus, the aim of this study was to investigate the safety of endoscopic ultrasound-guided CILA (EUS-CILA) performed using optimal settings in a porcine pancreas model with a two-week follow-up.

## 2. Methods

### 2.1. The Cylindrical Interstitial Laser Ablation Devices

**Figure 1:**
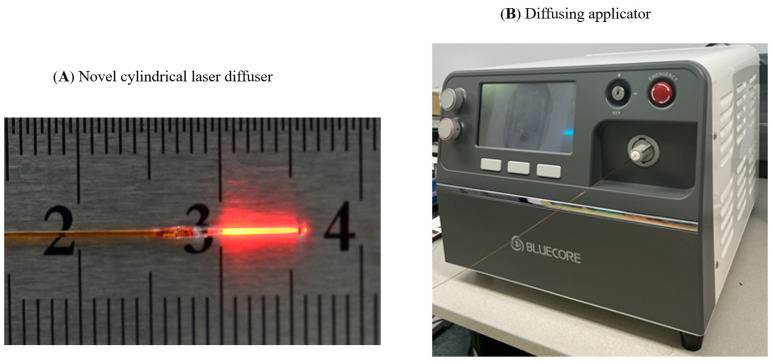
Laser ablation device.

The cylindrical laser diffuser (core diameter 400 µm; TeCure, Inc., Busan, Korea) was prepared using a micro-matched fiber tip surface. The 5 mm long active segment was crafted to emit laser light circumferentially (Figure 1A). Details of the fabrication procedure are provided in our previous study [13]. A 19-gauge needle (Cook Medical Inc., Bloomington, IN, USA) was used to puncture the pancreas under real-time EUS (EU-ME2 ultrasound system, GF-UCT260, Olympus Medical Co., Ltd., Tokyo, Japan) guidance, and the laser diffuser coupled to a customized 1064 nm Nd:YAG laser system (Bluecore Company, Busan, Korea) was inserted through the needle to reach the targeted area (Figure 1B).

### 2.2. Animal Models

Animal experiments were conducted according to the guidelines issued by the Korean National Institutes of Health, and the study protocol was approved by the Institutional Animal Care and Use Committee of Knotus, Korea (approval number: KNOTUS IACUC 21-KE-821). Five pigs (Sus scrofa domesticus, body weights between 30 and 35 kg) were used in the study. Before EUS-CILA, animals were anesthetized using 1:1 combination of zoletil (Virbac, Carros, France) and xylazine (Rompun; Bayer AG, Leverkusen, Germany). After intubation, general anesthesia was maintained using a mechanical ventilator providing isoflurane (1–2%) and oxygen (2 L/min). Entire procedures were performed with animals in the decubitus position, and a veterinarian monitored cardiopulmonary status throughout.

### 2.3. Endoscopic Ultrasound-Guided Laser Ablation

A linear-array EUS scope with a 19-gauge biopsy needle was inserted into the stomach using the EUS-guided trans-gastric approach. The biopsy needle was then advanced under EUS-guidance into the targeted pancreatic area, retrieved by 10 mm, and then the laser diffuser was inserted through the lumen of the biopsy needle. Based on our previous results, a laser energy of 450 J (5 W for 90 s) was applied [14]. Technical success was defined as successful completion of EUS-CILA at this energy setting.

### 2.4. Laboratory Test

Laboratory tests were performed at baseline, immediately after EUS-CILA, and a week and two weeks (before harvest) after EUS-CILA to evaluate organ damage and complications, including pancreatitis. White blood cell (WBC), red blood cell (RBC), and platelet (PLT) counts and aspartate aminotransferase (AST), alanine aminotransferase (ALT), alkaline phosphatase (ALP), γ-glutamyl transpeptidase (GGT), total bilirubin (T.Bil), blood urea nitrogen (BUN), creatinine, amylase, and lipase levels were determined at each time point.

### 2.5. CT Evaluation

A week after EUS-CILA, all animals were evaluated by computed tomography (CT; SOMATOM Emotion; Siemens Healthineers, Erlangen, Germany) using the following settings: slide thickness 3 mm, tube voltage 120 kV, and a tube current-exposure time product of 150 mAs. Procedure-related adverse events, including hemorrhage, pancreatitis, blood vessel injury, and organ perforation, were evaluated.

### 2.6. Necropsy and Histological Analysis

Pigs were sacrificed 2 weeks after EUS-CILA, and pancreases were removed by laparotomy. The efficacies and safeties of EUS-CILA were evaluated by necropsy (macroscopic analysis) and histological examination (microscopic analysis). Harvested pancreases were fixed in 10% formalin, cut transversely into 5-mm-thick slices, stained with hematoxylin and eosin (H&E), and scanned with a VENTANA iScan HT digital slide scanner (Roche Diagnostics, Sant Cugat, Spain). Tissue biological and structural changes were evaluated and photographed using QuPath software (an open-source software platform). Ablated areas in sections, measured using diameters of fibrotic changes, were used to calculate ablated volumes.

### 2.7. Statistical Analysis

The analysis was conducted using SPSS ver. 25 (SPSS Inc., Chicago, IL, USA). ANOVA was used to determine the significances of differences, which were accepted for *p* values of <0.05.

## 3. Results

### 3.1. Feasibility of EUS-Guided Ablation

In all animals, the pancreas was well visualized through the stomach using the linear EUS probe, and no difficulty or resistance was experienced during endoscope insertion. In each case, a 19-gauge needle was inserted and positioned into the targeted area. The cylindrical laser diffuser was inserted into the 19-gauge needle without bending or damaging and was well visualized as a hyperechoic line under real-time EUS guidance. During LA, a hyperechoic area was observed to increase around the tip of the diffuser (Video S1). No technical difficulty was experienced during any procedure, and all five pigs received predetermined doses of LA energy. Vital signs remained stable during procedures.

### 3.2. Safety Analysis and Laboratory Tests after CILA

All animals were observed for 2 weeks after EUS-CILA to evaluate complications. Major complications were mortality, peritonitis, sepsis, hemorrhage, cholangitis, and pancreatitis with clinical manifestations. At 2 weeks, no abnormal behavior, suggesting a major complication, was observed in any animal. WBC, Hb, and PLT were lower immediately after EUS-CILA than at baseline. PLT and BUN were higher a week after EUS-CILA, but ALP was lower at one and two weeks than at baseline. Mean amylase levels were 1.79 and 2.86 times baseline at one and two weeks. However, none of these changes were clinically significant (Table 1). These results indicate inflammatory marker levels, including those of acute pancreatitis, were not elevated after EUS-CILA. Furthermore, body weight of porcine were not change during the study period without any significant change in feed intake indicates that our CILA treatment did not affect the function of pancreas (Figure S1).

### 3.3. CT and Necropsy

**Figure 2:**
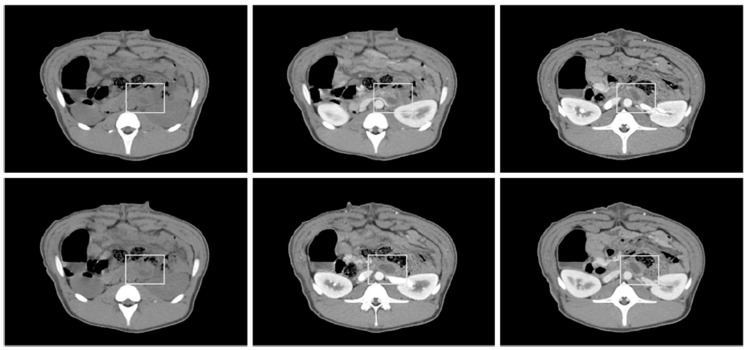
Computed tomography non-contrast (left) image and early (center) and delayed (right) phases of contrast-enhanced CT images: Images showed no evidence of perforation, hemorrhage, ascites, adjacent structural injury, or peri-pancreatic fluid collection. Images showed a solid, low-density lesion in EUS-CILA-treated region (white rectangles).

CT showed no evidence of perforation, hemorrhage, ascites, adjacent structural injury, or peri-pancreatic fluid collection. Images showed a solid, low-density lesion in EUS-CILA-treated regions of pancreas bodies and no expansion of pancreatic contours or inflammatory changes in peripancreatic fat (Figure 2). Pancreases and surrounding organs were grossly examined during harvest laparotomy at 2 weeks after EUS-CILA, and no gross abnormalities such as peri-pancreatic fluid collection, perforation, hematoma, adhesion to adjacent structures, or surrounding organ injuries were observed.

### 3.4. Tissue Analysis

**Figure 3:**
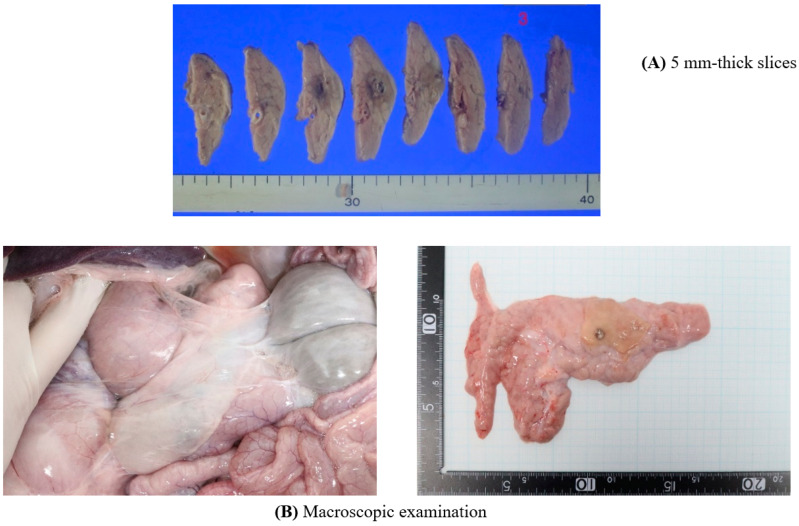
Macroscopic examination Assessments of thermal ablation in in vivo pancreatic tissue 2 weeks after LA: macroscopic examination image revealed no gross abnormalities, such as perforation, adhesion to adjacent structures, hematoma to surrounding organs in any of the five pigs.

Gross pathological specimens from 5-mm-thick pancreatic slices were used to assess degrees of CILA-induced coagulation. Ablated areas were well distinguished as dark brown lesions surrounded by normal pancreatic tissue (Figure 3A,B). A uniform circular area of necrosis with surrounding fibrosis was observed in hematoxylin and eosin-stained sections (Figure 3B). Moreover, loss of normal pancreatic acinar cell structures in treated areas with surrounding fibrosis suggested complete cell death by necrosis by ablation. Focal neutrophilic abscesses and macrophage aggregations were observed in each case, indicating that rapid and transient inflammation had occurred in ablated areas. Microscopic examination confirmed the presence of necrosis in treated pancreatic tissues due to clear demarcation between CILA-ablated areas and untreated tissues. In four pigs, focal microscopic perforations related to thermal injury were observed on abutting small intestine.

**Figure 4:**
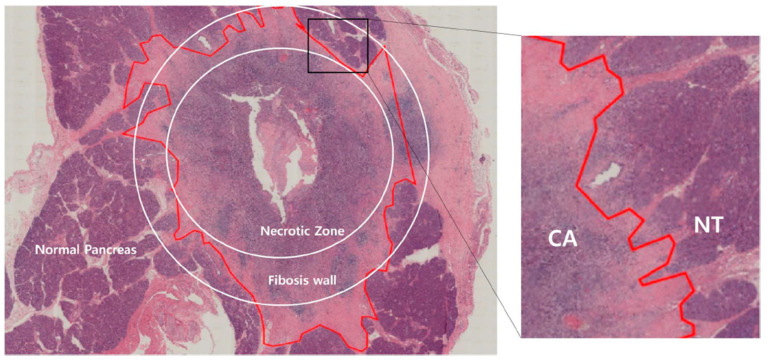
Microscopic examination Histological image of the laser ablation outcome: Untreated parenchyma and ablated tissues were clearly differentiated with coagulated necrosis and fibrous tissue area on H&E-staining. NT: normal tissue, CA: coagulative area (40×).

**Figure 5:**
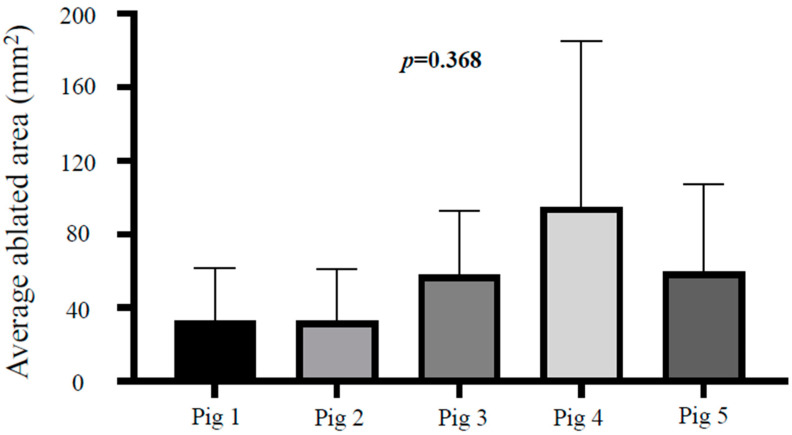
Average fibrosis area after EUS-CILA Mean ablated areas in the pancreatic sections of the five pigs were not significantly different (*p* = 0.368).

EUS-CILA ablated regions and untreated parenchyma were clearly differentiated in H&E-stained specimens (Figure 4). Mean ablated volume (cross-sectional area × length), as determined by histologic examination, was 55.5 mm^2^ × 29.0 mm, and mean ablated areas in the pancreatic sections of the five pigs were not significantly different (*p* = 0.368) (Figure 5).

## 4. Discussion

This two-week follow-up study after EUS-CILA, performed using optimal settings, demonstrates that EUS-CILA is technically feasible, effective, and relatively safe when applied to porcine pancreas. In a previous study, we observed acute responses in porcine pancreatic tissue 3 days after EUS-guided CILA without any notable toxicity related to laser ablative treatment. Our CILA has demonstrated the potential to overcome the shortcomings of conventional laser fibers that emit light through an end-firing unidirectional diffuser that does not achieve uniform tissue coagulation and presents the risk of thermal damage to surrounding normal tissues or adjacent organs [12,15]. However, although the procedure was technically successful, the study was limited by its short follow-up. Therefore, in the present study, we evaluated and confirmed the efficacy and safety of EUS-CILA using a 2-week follow-up. In addition, we also confirmed that EUS-CILA causes uniform areas of necrosis in porcine pancreatic tissues when administered using the same energy settings. To the best of our knowledge, this is the first study to study the in vivo effects of EUS-CILA in pancreas with a relatively long follow-up.

In the majority of patients with unresectable pancreatic cancer at diagnosis, therapeutic options are limited, and prognosis is dismal. Local ablative treatment in pancreatic cancer offers promise in terms of improving clinical outcomes and has been used to treat prostate, liver, and thyroid tumors [16,17]. Several non-randomized studies have suggested adding local ablation treatment for patients with LAPC has survival benefit [6,18]. Moreover, additional local ablation treatment may elicit an antitumor immune response by increasing the availability of tumor-specific neoantigens in inflammatory situations, which suggests this type of treatment has the potential to help overcome resistance to immunotherapies in pancreatic cancer [19]. The use of thin optic fibers enables the treatment of pancreatic cancers that are difficult to approach but could damage surrounding normal organs. EUS-guided local ablative treatment has been attempted for selective tissue ablation to minimize damage to surrounding tissues, and EUS-guided ablation therapies have been widely attempted as alternative pancreatic cancer treatments. EUS provides an effective means of targeting pancreatic tumors [20], and EUS-RFA is one of the local ablative treatment modalities most studied for the control of locally advanced pancreatic cancer. Unfortunately, the technique can be harmful because its related complications range from moderate pancreatitis to peritonitis [21,22]. On the other hand, LA requires a fine optic fiber rather than an RFA needle, and thus overcomes the shortcomings of RFA and provides safe access to deep organs with a complex anatomy that cannot be reached by RFA without causing mechanical injuries [23]. Furthermore, pancreatic tissue is sensitive, and thus, local ablative treatment could easily damage its endocrine and exocrine functions. For this reason, safety is a primary concern of those undertaking local ablative treatment for pancreatic cancer.

In the present study, mean ablated area (area × length) was 55.5 mm^2^ × 29.0 mm at 450 J (5 W for 90 s), and mean ablated areas in sections in porcine pancreases were similar, which confirmed our previous result that EUS-CILA predictably generates uniform ablation in porcine pancreatic tissue. The first clinical study on EUS-LA conducted on nine patients with locally advanced pancreatic cancer highlighted several shortcomings that required resolution [24]. Specifically, ablated volumes varied from 0.4 to 6.3 cm^3^ and no relationship was observed between administered LA energy and ablated volume. In addition, the follow-up period was relatively short, and thus long-term efficacy was not addressed. Other clinical studies on the use of LA for pancreatic cancer are underway, but no results have been reported. Furthermore, no preclinical or clinical study has reported long-term outcomes. Therefore, we undertook this study to investigate response to EUS-CILA using a 2-week follow-up and found a consistent relationship between energy dose and affected volume. We also evaluated long-term histologic changes after EUS-CILA, which have not been previously examined though several studies have investigated short-term histologic changes [11,25,26]. Our findings show that EUS-CILA-induced histological pancreatic changes persist for at least 2 weeks.

Furthermore, no mortality, major complication, or adverse outcome was observed during the experimental period, which confirmed the results we obtained during in a previous short-term experiment [14]. In the current study, we used a follow-up of 2 weeks to accurately determine the long-term safety of EUS-CILA [25,26]. The only clinical trial conducted on EUS-guided laser ablation for pancreatic cancer (excluding case series) reported no major event during LA in all nine patients included. However, post-procedural fluid collection surrounding the pancreas was observed in three patients (33%), more than a threefold increase in serum amylase was noted in two (22%), and one patient died of a non-procedurally related complication before a scheduled 30-day CT scan [24]. In contrast, in the present study, only minimal complications were encountered during or after EUS-CILA, and no fluid collection or meaningful adjacent organ damage was observed by CT or histologic analysis.

The present study has a number of limitations that warrant consideration. First, because this study was conducted using a porcine model, our results may not accurately reflect results in humans because of the differences between cancer microenvironments, which are composed of dense networks of fibroblasts and infiltrating immune and endothelial cells. Second, the trans-gastric approach was used, which did not allow adequate visualization of the pancreatic head but enabled excellent visualization of the pancreatic body and tail. Unfortunately, the trans-duodenal approach, which would have provided visualization of the pancreatic head was not feasible because stomachs are larger, and pyloric muscle is thicker in pigs than in man. Finally, only five pigs were included, and thus, we suggest a larger-scale study animal study be conducted to confirm our findings before clinical applications.

## 5. Conclusions

In conclusion, our findings suggest that EUS-CILA is feasible and a potentially effective local treatment modality for pancreatic cancer. The diffusing applicator provides a uniform circular ablation area. Notably, there was no major morbidity or mortality during the study period. We believe CILA could offer safety as well as efficacy for pancreatic cancer patients.

## Figures and Tables

**Table 1:** Blood test results after EUS-CILA.

Variables ^§^	Baseline	Immediately after CILA	*p*-Value	1 Week after CILA	*p*-Value	2 Weeks after CILA	*p*-Value
WBC (×10^3^ cells/μL)	20.48	19.35	0.03	19.39	0.91	19.93	0.99
Hb (g/dL)	11.78	11.26	0.02	10.96	0.07	11.04	0.05
PLT (×10^3^ cells/μL)	416.80	382.00	0.04	597.40	0.03	436.60	0.84
AST	34.54	36.92	0.79	27.46	0.14	24.94	0.08
ALT	37.52	36.58	0.25	33.00	0.34	36.92	1.00
ALP	395.06	386.86	0.67	249.82	0.05	251.08	0.05
GGT	40.26	41.50	0.18	41.00	0.96	36.27	0.10
T.Bil	0.27	0.25	0.81	0.26	0.97	0.28	1.00
BUN	11.85	11.83	1.00	13.08	0.00	10.74	0.40
Creatinine	1.48	1.46	0.99	1.49	1.00	1.53	0.96
Amylase	1366.20	1304.00	0.15	1390.40	0.99	1401.20	0.99
Lipase	8.36	7.66	0.06	14.98	0.48	23.92	0.49

Abbreviation: WBC, White blood cell count; Hb, hemoglobin; PLT, platelet count; AST, aspartate aminotransferase; ALT, alanine aminotransferase; ALP, alkaline phosphatase; GGT, gamma-glutamyl transferase; T.Bil, total bilirubin; BUN, blood urea nitrogen; ^§^, mean.

## Data Availability

The data presented in this study are available on request from the corresponding author. The data are not publicly available due to privacy or ethical restrictions.

## Supplementary Materials

The following supporting information can be downloaded at: https://www.mdpi.com/article/10.3390/biomedicines10112895/s1. Video S1. EUS-CILA of in vivo pancreas under EUS guidance, the CILA diffuser was clearly visible during the application of laser energy to the target lesion. The hyperechoic lesion around the tip of the diffuser gradually increased in size according to the delivered laser energy. Figure S1: Body weight changes during experiment.

## Abbreviations

LAPC: locally advanced pancreatic cancer; LA, laser ablation; RFA, radiofrequency ablation; EUS, endoscopic ultrasonography; Nd:YAG, neodymium-doped yttrium aluminum garnet; CT, computed tomography.

## References

[1] Sung H., Ferlay J., Siegel R.L., Laversanne M., Soerjomataram I., Jemal A., Bray F. (2021). Global Cancer Statistics 2020: GLOBOCAN Estimates of incidence and mortality worldwide for 36 cancers in 185 countries. CA Cancer J. Clin..

[2] Ferlay J., Partensky C., Bray F. (2016). More deaths from pancreatic cancer than breast cancer in the EU by 2017. Acta Oncol..

[3] Paniccia A., Edil B.H., Schulick R.D., Byers J.T., Meguid C., Gajdos C., McCarter M.D. (2014). Neoadjuvant FOLFIRINOX application in borderline resectable pancreatic adenocarcinoma: A retrospective cohort study. Medicine.

[4] Kim Y., Song K., Lee Y., Park K., Hwang D., Lee J., Shin S., Kwon J., Ro J., Kim S. (2019). Management of isolated recurrence after surgery for pancreatic adenocarcinoma. Br. J. Surg..

[5] Huguet F., Girard N., Guerche C.S.-E., Hennequin C., Mornex F., Azria D. (2009). Chemoradiotherapy in the management of locally advanced pancreatic carcinoma: A qualitative systematic review. J. Clin. Oncol..

[6] Keane M.G., Bramis K., Pereira S.P., Fusai G.K. (2014). Systematic review of novel ablative methods in locally advanced pancreatic cancer. World J. Gastroenterol..

[7] Cazacu I.M., Singh B.S., Saftoiu A., Bhutani M.S. (2020). Endoscopic ultrasound-guided treatment of pancreatic cancer. Curr. Gastroenterol. Rep..

[8] White R.R., Murphy J.D., Martin R.C. (2021). The Landmark Series: Locally Advanced Pancreatic Cancer and Ablative Therapy Options. Ann. Surg. Oncol..

[9] Schena E., Saccomandi P., Fong Y. (2017). Laser ablation for cancer: Past, present and future. J. Funct. Biomater..

[10] Di Matteo F., Martino M., Rea R., Pandolfi M., Panzera F., Stigliano E., Schena E., Saccomandi P., Silvestri S., Pacella C.M. (2013). US-guided application of Nd: YAG laser in porcine pancreatic tissue: An ex vivo study and numerical simulation. Gastrointest. Endosc..

[11] Di Matteo F., Martino M., Rea R., Pandolfi M., Rabitti C., Masselli G.M.P., Silvestri S., Pacella C.M., Papini E., Panzera F. (2010). EUS-guided Nd: YAG laser ablation of normal pancreatic tissue: A pilot study in a pig model. Gastrointest. Endosc..

[12] Lee J., Lee S., Truong V.G., Lim S., Kang H.W., Jung J.H., Park J.-S. (2022). Laser ablation of pancreatic cancer using a cylindrical light diffuser. Lasers Med. Sci..

[13] Truong V.G., Park S., Kang H.W. (2017). Spatial effect of conical angle on optical-thermal distribution for circumferential photocoagulation. Biomed. Opt. Express.

[14] Lim S., Truong V.G., Choi J., Jeong H.J., Oh S.-J., Park J.-S., Kang H.W. (2022). Endoscopic Ultrasound-Guided Laser Ablation Using a Diffusing Applicator for Locally Advanced Pancreatic Cancer Treatment. Cancers.

[15] Lee J., Park H.Y., Kim W.W., Jeong J.Y., Lee Y.-D., Choi M.-H., Kim S., Park J.-Y., Jung J.H. (2017). Combination treatment with photodynamic therapy and laser ablation in breast cancer: An animal model study. Photomed. Laser Surg..

[16] Francica G., Petrolati A., Di Stasio E., Pacella S., Stasi R., Pacella C.M. (2012). Effectiveness, safety, and local progression after percutaneous laser ablation for hepatocellular carcinoma nodules up to 4 cm are not affected by tumor location. AJR Am. J. Roentgenol..

[17] Pacella C., Mauri G., Achille G., Barbaro D., Bizzarri G., De Feo P., Di Stasio E., Esposito R., Gambelunghe G., Misischi I. (2015). Outcomes and risk factors for complications of laser ablation for thyroid nodules: A multicenter study on 1531 patients. J. Clin. Endocrinol. Metab..

[18] Hua Y.-Q., Wang P., Zhu X.-Y., Shen Y.-H., Wang K., Shi W.-D., Lin J.-H., Meng Z.-Q., Chen Z., Chen H. (2017). Radiofrequency ablation for hepatic oligometastatic pancreatic cancer: An analysis of safety and efficacy. Pancreatology.

[19] Gajiwala S., Torgeson A., Garrido-Laguna I., Kinsey C., Lloyd S. (2018). Combination immunotherapy and radiation therapy strategies for pancreatic cancer—targeting multiple steps in the cancer immunity cycle. J. Gastrointest. Oncol..

[20] Song T.J., Seo D.W., Lakhtakia S., Reddy N., Oh D.W., Park D.H., Lee S.S., Lee S.K., Kim M.-H. (2016). Initial experience of EUS-guided radiofrequency ablation of unresectable pancreatic cancer. Gastrointest. Endosc..

[21] Carrara S., Arcidiacono P.G., Albarello L., Addis A., Enderle M., Boemo C., Campagnol M., Ambrosi A., Doglioni C., Testoni P.A. (2008). Endoscopic ultrasound-guided application of a new hybrid cryotherm probe in porcine pancreas: A preliminary study. Endoscopy.

[22] Gaidhane M., Smith I., Ellen K., Gatesman J., Habib N., Foley P., Moskaluk C., Kahaleh M. (2012). Endoscopic ultrasound-guided radiofrequency ablation (EUS-RFA) of the pancreas in a porcine model. Gastroenterol. Res. Pract..

[23] Li W., Liu J., Huang T.Y., Zhong X., Yang D.P., Xie X.H., Liu D.H., Xie X.Y., Zhuang B.W. (2020). Lesion outline and thermal field distribution of ablative in vitro experiments in myocardia: Comparison of radiofrequency and laser ablation. BMC Cardiovasc. Disord..

[24] Di Matteo F.M., Saccomandi P., Martino M., Pandolfi M., Pizzicannella M., Balassone V., Schena E., Pacella C.M., Silvestri S., Costamagna G. (2018). Feasibility of EUS-guided Nd: YAG laser ablation of unresectable pancreatic adenocarcinoma. Gastrointest. Endosc..

[25] Truong V.G., Jeong S., Park J.-S., Kim S.M., Lee D.H., Kang H.W. (2021). Endoscopic ultrasound (EUS)-guided cylindrical interstitial laser ablation (CILA) on in vivo porcine pancreas. Biomed. Opt. Express.

[26] Truong V.G., Kim H., Park J.-S., Tran V.N., Kang H.W. (2021). Multiple cylindrical interstitial laser ablations (CILAs) of porcine pancreas in ex vivo and in vivo models. Int. J. Hyperth..

