# Supplementary figures and images for: Endoscopic Ultrasound-Guided Pancreatic Interstitial Laser Ablation Using a Cylindrical Laser Diffuser: A Long-Term Follow-Up Study
Source: Biomedicines. 2022 Nov 11;10(11):2895. doi: 10.3390/biomedicines10112895 (PMC9687491; doi:10.3390/biomedicines10112895)

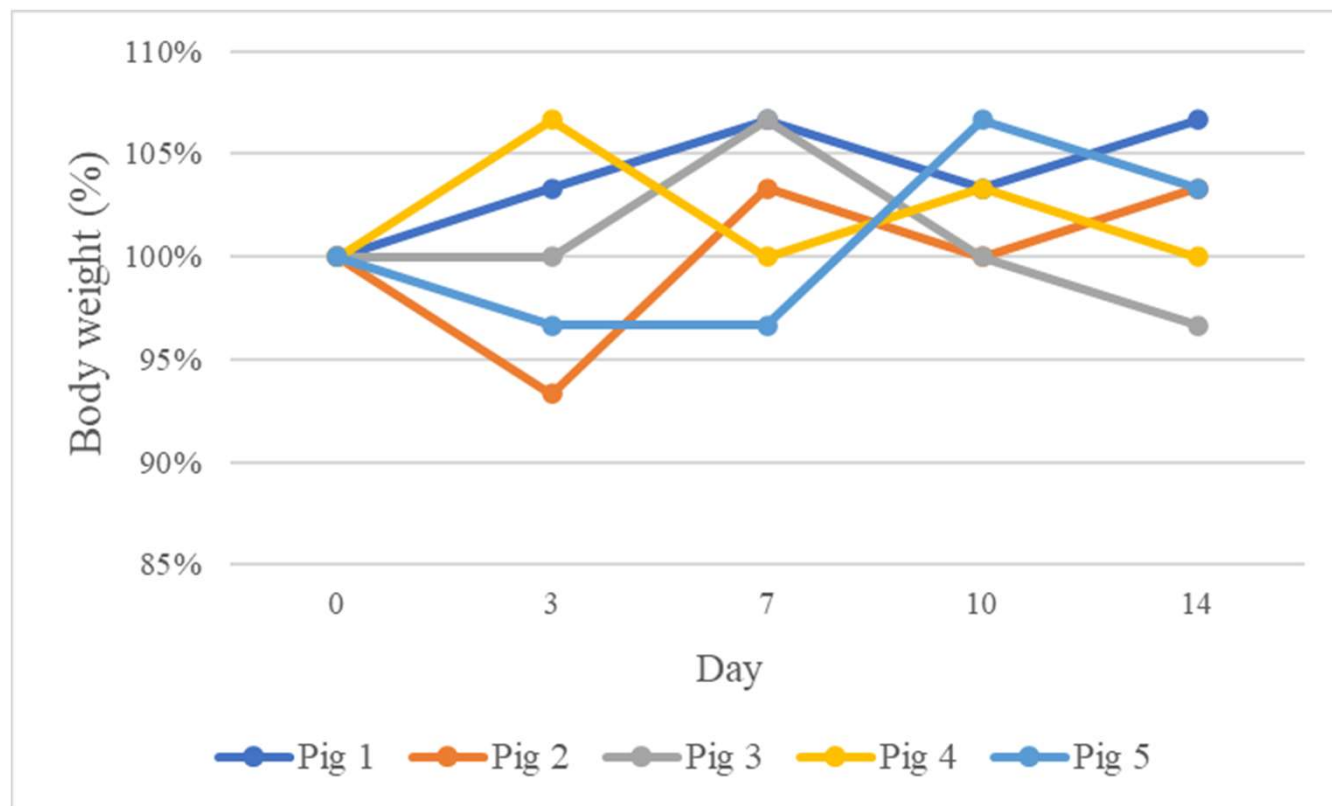

Supplement: Supplementary file 1 [file biomedicines-10-02895-s001.zip › biomedicines-2009048 sup/biomedicines-2009048 figure S1.pdf]
